# Peretinoin, an acyclic retinoid, inhibits hepatocarcinogenesis by suppressing sphingosine kinase 1 expression *in vitro* and *in vivo*

**DOI:** 10.1038/s41598-017-17285-2

**Published:** 2017-12-05

**Authors:** Masaya Funaki, Juria Kitabayashi, Tetsuro Shimakami, Naoto Nagata, Yuriko Sakai, Kai Takegoshi, Hikari Okada, Kazuhisa Murai, Takayoshi Shirasaki, Takeru Oyama, Taro Yamashita, Tsuguhito Ota, Yoh Takuwa, Masao Honda, Shuichi Kaneko

**Affiliations:** 1 Department of Gastroenterology, Kanazawa University, Kanazawa, Ishikawa, Japan; 2 Advanced Preventive Medical Sciences Research Center, Kanazawa University, Kanazawa, Ishikawa, Japan; 3 Department of Molecular and Cellular Pathology, Graduate School of Medical Science, Kanazawa University, Ishikawa, Japan; 4 Department of Physiology, Kanazawa University School of Medicine, Kanazawa, Ishikawa, Japan

## Abstract

Sphingosine-1-phospate is a potent bioactive lipid metabolite that regulates cancer progression. Because sphingosine kinase 1 and sphingosine kinase 2 (SPHK 1/2) are both essential for sphingosine-1-phospate production, they could be a therapeutic target in various cancers. Peretinoin, an acyclic retinoid, inhibits post-therapeutic recurrence of hepatocellular carcinoma via unclear mechanisms. In this study, we assessed effects of peretinoin on SPHK expression and liver cancer development *in vitro* and *in vivo*. We examined effects of peretinoin on expression, enzymatic and promoter activity of SPHK1 in a human hepatoma cell line, Huh-7. We also investigated effects of SPHK1 on hepatocarcinogenesis induced by diethylnitrosamine using SPHK1 knockout mice. Peretinoin treatment of Huh-7 cells reduced mRNA levels, protein expression and enzymatic activity of SPHK1. Peretinoin reduced SPHK1 promoter activity; this effect of peretinoin was blocked by overexpression of Sp1, a transcription factor. Deletion of all Sp1 binding sites within the SPHK1 promoter region abolished SPHK1 promoter activity, suggesting that peretinoin reduced mRNA levels of SPHK1 via Sp1. Additionally, diethylnitrosamine-induced hepatoma was fewer and less frequent in SPHK1 knockout compared to wild-type mice. Our data showed crucial roles of SPHK1 in hepatocarcinogenesis and suggests that peretinoin prevents hepatocarcinogenesis by suppressing mRNA levels of SPHK1.

## Introduction

Worldwide, hepatocellular carcinoma (HCC) is the fifth most common cancer and the second most common cause of cancer death, estimated to be responsible for 745,000 deaths in 2012^1^. Infection with hepatitis B virus and/or hepatitis C virus (HCV) is a major cause of HCC^2–4^. Due to dramatic progress in antiviral treatments, hepatitis viral infection is now much easier to control. In particular, highly efficient direct-acting antiviral agents can eliminate HCV from the infected liver in more than 90% of cases^5^. However, considerable time is required for the liver to fully recover from liver cirrhosis and HCC is reported to emerge from HCV-infected livers at a rate of about 1% per year, even after HCV is successfully eliminated^6–8^.

HCC can be treated by means of ablation, surgical resection, catheter embolization, chemotherapy, and liver transplantation, depending on HCC stage and liver function. Curative resection or ablation is applied to early-stage HCC but the 3-year recurrence rate after curative treatment in the general population is 50%^9,10^. Moreover, the recurrence rate is >70% in HCV-infected patients^11^. Therefore, new therapeutic strategies to prevent HCC recurrence or hepatocarcinogenesis are urgently needed.

Peretinoin (generic name code: NIK-333), which was developed by Kowa Company, Ltd. (Aichi, Japan), is an orally available acyclic retinoid with a vitamin A-like structure that targets retinoid nuclear receptors, such as retinoid X receptor (RXR) and retinoic acid receptor (RAR)^12^. Administration of peretinoin significantly reduces the incidence of post-therapeutic HCC recurrence and improves patient survival^13–15^. In addition, peretinoin prevents the development of HCC in several mouse models^16–18^. Currently, larger-scale clinical studies are ongoing in various countries to confirm its clinical efficacy in preventing HCC after curative treatment.

The mechanisms underlying hepatocarcinogenesis prevention by peretinoin have been extensively studied. We reported that peretinoin prevented hepatocarcinogenesis, as well as hepatic fibrosis and steatosis, in a PDGF-C transgenic (PDGF-C Tg) mouse model^16^. We also recently reported that peretinoin prevented progression of non-alcoholic steatohepatitis (NASH) and HCC development by activating the autophagy pathway in an atherogenic and high-fat (Ath-HF) diet mouse model^17^. In these studies, we also performed comprehensive gene expression analysis in the mouse liver with a microarray technique, which revealed that the expression of sphingosine kinase 1 (SPHK1) was significantly upregulated in the PDGFC-Tg mice and Ath-HF diet mice compared with control mice and that this increase was suppressed by peretinoin. Therefore, we hypothesized that peretinoin could prevent hepatocarcinogenesis by modifying sphingolipid metabolism.

SPHK exists in two isoforms, SPHK1 and SPHK2, both of which phosphorylate sphingosine to generate sphingosine-1-phospate (S1P). Several lines of evidence indicate that S1P is an important regulator of many cancers, including prostate, colon, breast, and gastric cancer, by promoting several aspects, such as transformation, metastasis, neovascularization, angiogenesis, and resistance to anti-cancer agents^19–22^. Therefore, SPHK1 and SPHK2, as well as S1P itself, have been recognized as promising targets for cancer treatment, and many SPHK inhibitors, either selective or non-selective for SPHK1 and SPHK2, have been developed, some of which are now being used in clinical trials^23,24^. Anti-S1P monoclonal antibody has also been developed, and its effectiveness in several cancers has been shown by using *in vitro* models^25^. However, the importance of the SPHK–S1P axis in hepatoma is poorly understood compared with other cancers.

In this study, we demonstrate that peretinoin reduces the mRNA level of SPHK1 *in vitro* by downregulating a transcription factor, Sp1. Additionally, diethylnitrosamine (DEN)-induced hepatoma was significantly fewer and less frequent in SPHK1 knockout mice compared to wild-type mice Collectively, our results suggest that one of the mechanisms by which peretinoin prevents hepatocarcinogenesis would be suppression of SPHK1 expression and that the SPHK–S1P axis could be a promising target for HCC therapy.

## Results

### High levels of SPHK1 mRNA in the liver in two different mouse hepatoma models

First, we investigated how peretinoin could prevent hepatocarcinogenesis by using two types of mouse liver fibrosis-hepatoma models, a PDGF-C Tg model^16,26^ and an atherogenic and high-fat (Ath + HF) diet model^27^. Briefly, in the PDGF-C Tg model mouse, overexpression of PDGF-C in mouse liver results in hepatic fibrosis, steatosis, and ultimately HCC; this model is quite similar to human HCC, in that HCC usually develops from fibrotic liver. In the Ath + HF diet model mouse, the diet induces non-alcoholic steatohepatitis and eventual development of HCC. We previously demonstrated a suppressive effect of peretinoin on hepatocarcinogenesis in both of these models and performed a comprehensive gene expression analysis on non-cancerous regions of the liver by using a cDNA microarray technique^16,17^.

**Figure 1:**
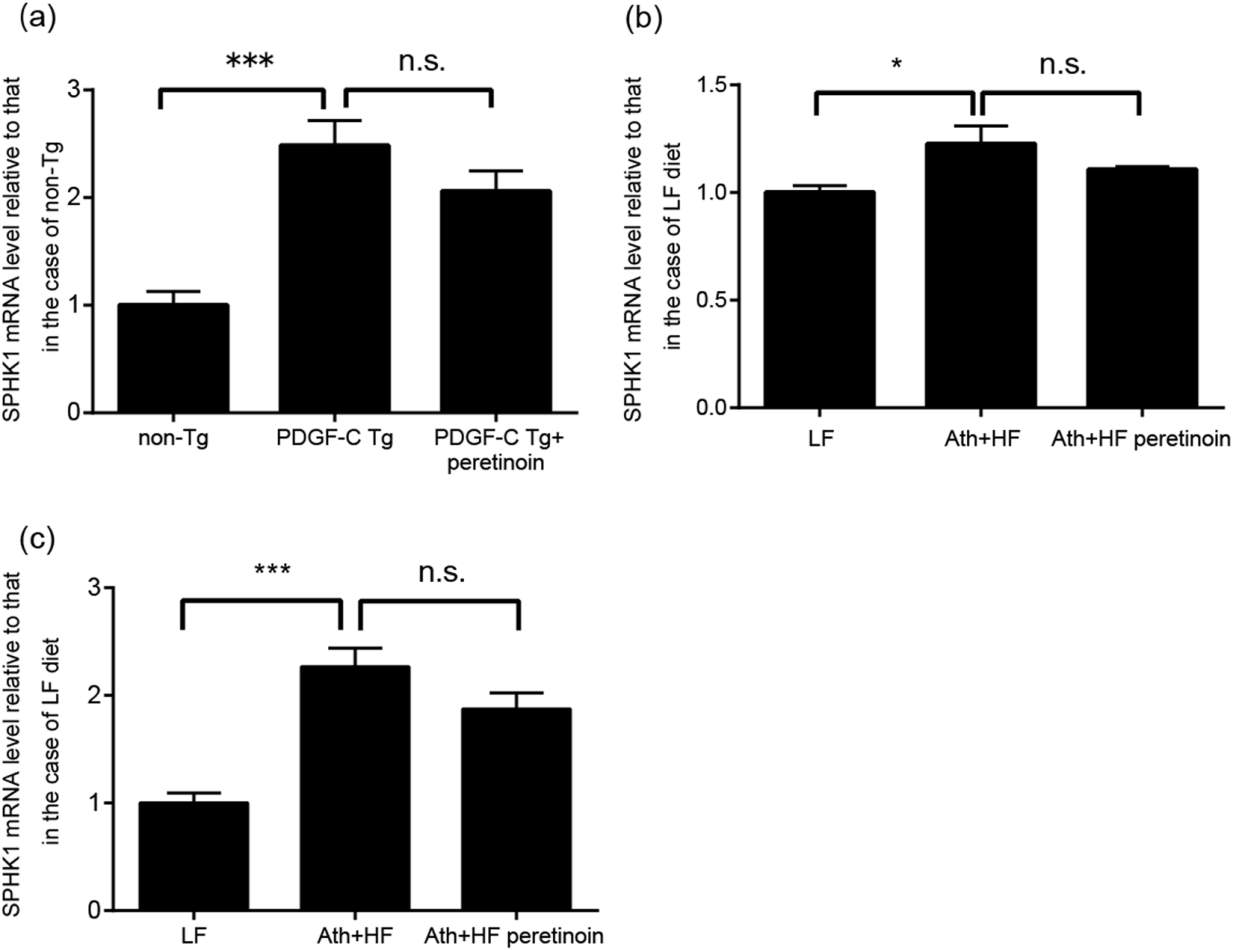
Liver mRNA levels of SPHK1 in the PDGFC-Tg and Ath-HF diet mouse models. (**a**) mRNA level of SPHK1 in the liver of PDGF-C Tg mice determined by microarray analysis. We normalized the mRNA level of SPHK1 in the liver of PDGF-C Tg mice treated with or without 0.06% peretinoin to that of non-Tg mice. The mRNA levels were calculated from the microarray data published in one of our previous reports^16^. This figure shows the relative mRNA level of SPHK1 under each condition to that of non-Tg mice. Error bars indicate the standard deviation from three mice. The statistical significance of the difference in the average between the two groups was analyzed by the Student’s t test. (**b**) mRNA levels in the liver in the Ath-HF diet mouse model determined by microarray analysis. We normalized the mRNA level of SPHK1 in the liver of Ath-HF diet mice with or without 0.03% peretinoin to that of LF mice. The mRNA levels were calculated from the microarray data published in one of our previous reports^17^. This figure shows the relative mRNA level of SPHK1 under each condition to that in LF mice. Error bars indicate the standard deviation from three mice. The statistical significance was analyzed as above. (**c**) mRNA level of the liver in the Ath-HF diet mouse model determined by qRT-PCR. We quantitated the mRNA levels of SPHK1 and β-actin in the liver of Ath-HF diet mice with or without 0.03% peretinoin to that of LF mice by qRT-PCR (TaqMan assay); the mRNA level of SPHK1 was then normalized to that of β-actin for each mouse. The normalized mRNA levels of SPHK1 in the liver of Ath-HF diet mice with or without peretinoin were further normalized to that of LF mice. This figure shows the relative mRNA level of SPHK1 of each condition to that of LF mice. Error bars indicate the standard deviation from at least 10 mice. Statistical significance was analyzed as above. *p < 0.05, ***p < 0.005.

In this study, we analyzed the data obtained from those two studies by focusing on sphingolipid-related genes in a more specific manner. The signal intensity of SPHK1 mRNA in the non-cancerous liver derived from PDGF-C Tg mice was significantly higher than that from non-transgenic mice, whereas the high level of SPHK1 mRNA decreased in the mice treated with peretinoin to the level of the non-transgenic mice (Fig. 1a). The signal intensity of SPHK1 mRNA in the non-cancerous liver of the mice fed the Ath + HF diet was also significantly increased compared with that from mice fed the low-fat (LF) diet, but the high level of SPHK1 mRNA decreased in the mice treated with peretinoin, reaching that of the mice fed the LF diet (Fig. 1b). The mRNA level of SPHK1 in the mouse liver fed the Ath-HF diet measured by quantitative real-time PCR (qRT-PCR) was found to be identical to that determined by the microarray analysis (Fig. 1c).

### mRNA levels of other sphingolipid-related genes in the liver from two different mouse hepatoma models

By using data from the microarray analysis, we also examined other sphingolipid-related genes such as SPHK2 and S1P lyase, which degrade S1P and S1P receptor 1, S1P_1_. The signal intensity of SPHK2 mRNA was lower in the liver of mice fed the Ath-HF diet than in the liver of mice fed the LF diet, with peretinoin restoring the SPHK2 mRNA levels. The signal intensity of S1P lyase mRNA in the liver of mice fed the Ath + HF diet increased compared with that from the liver of mice fed the LF diet, but the increase in the S1P lyase mRNA was blunted by peretinoin treatment. On the other hand, the signal intensity of S1P_1_ was not altered by the Ath-HF diet or by peretinoin compared with the LF diet (supplemental Fig. S1a). We also did not detect any significant changes in SPHK2, S1P lyase, or S1P_1_ in PDGF-C Tg mice compared with non-transgenic mice (supplemental Fig. S1b). These results from two different mouse hepatoma models strongly suggest that SPHK1 plays a crucial role in hepatocarcinogenesis and that peretinoin could suppress SPHK1 expression.

### Analysis of SPHK1 mRNA levels in human liver based on liver fibrosis

**Figure 2:**
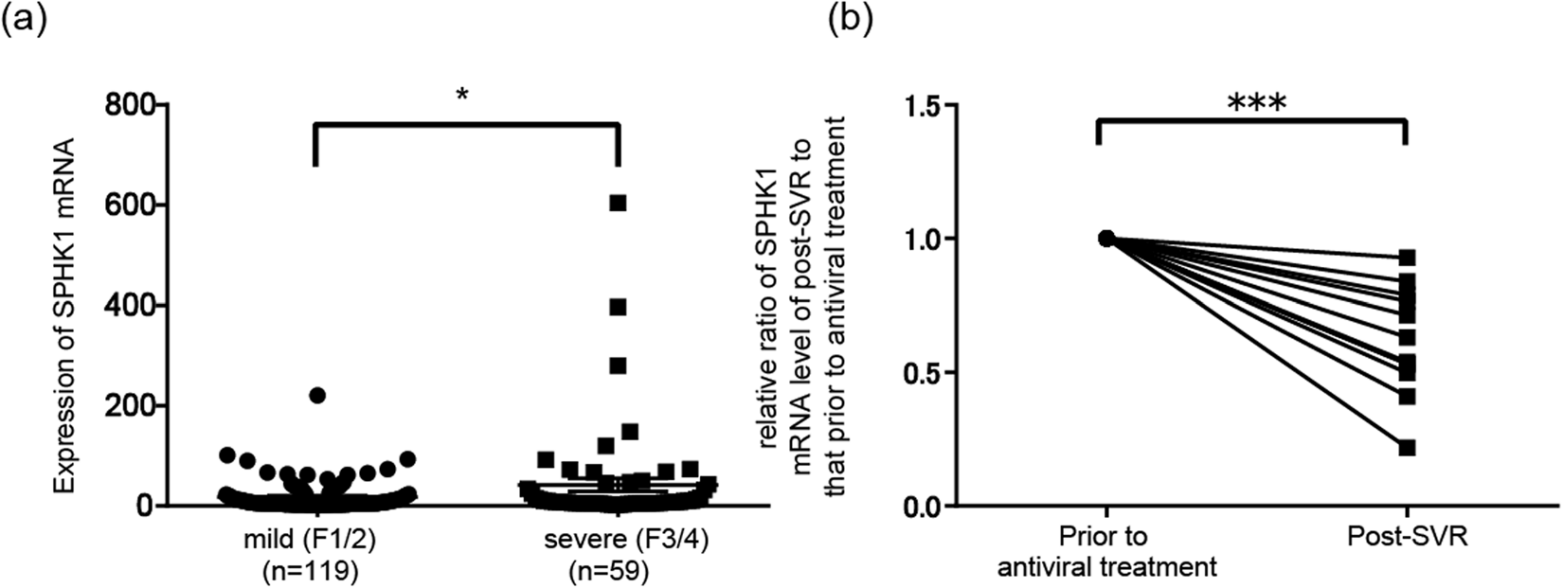
mRNA level of SPHK1 in human liver. **A**. comparison of the mRNA level of SPHK1 between mild and severe fibrosis in the HCV-infected liver. (**a**) We performed liver biopsies on patients infected with HCV prior to antiviral treatment and quantified the mRNA levels of SPHK1 and β-actin in total RNA extracted from the liver by qRT-PCR (TaqMan assay); the mRNA level of SPHK1 was then normalized to that of β-actin for each patient. The patients were categorized into two groups based on their liver fibrosis grade; mild fibrosis was defined as METAVIR Score F1 or F2 and severe fibrosis as F3 or F4. We compared the normalized mRNA level of SPHK1 between mild and severe fibrotic liver. Error bars show the standard deviation from 119 patients with mild liver fibrosis and 59 patients with severe fibrosis. The statistical significance of the difference in the average between the two groups was analyzed by the Student’s t test. (**b**) Comparison of the mRNA level of SPHK1 before and after HCV eradication. We performed liver biopsies on another 12 patients before they started antiviral treatment for HCV and after successful eradication of HCV. We quantitated the mRNA levels of SPHK1 and β-actin in total RNA extracted from the liver by qRT-PCR (TaqMan assay); the mRNA level of SPHK1 was then normalized to that of β-actin for each patient. The relative mRNA level of SPHK1 after HCV eradication (post-SVR [sustained virologic response]) was normalized to that before antiviral treatment (prior to antiviral treatment) in the individual patients, with the mRNA level of SPHK1 before antiviral treatment set to 1. The statistical significance of the difference in the average between these two groups was analyzed by a paired t test. *p < 0.05, ***p < 0.005.

Sato *et al*.^28^ recently showed that the mRNA level of SPHK1 was higher in severe fibrotic human liver than in mild fibrotic human liver. Here, we measured the mRNA levels of SPHK1 in human liver from 178 patients by qRT-PCR. All patients had HCV infection and liver samples were obtained prior to antiviral treatments. The degree of liver fibrosis was assessed by histologists and liver fibrosis was diagnosed based on the METAVIR score. Patients were categorized into two groups based on the score: F1 and F2 as mild fibrosis and F3 and F4 as severe fibrosis. When we compared the SPHK1 mRNA levels of these two groups, the level was significantly higher in the liver with severe fibrosis than in the liver with mild fibrosis (Fig. 2a).

### Decrease in the mRNA level of SPHK1 in the liver after HCV eradication

Next, we compared the mRNA levels of SPHK1 in liver tissues obtained prior to antiviral treatment for HCV and those obtained after successful eradication of HCV by the treatment. The clinical background and type of antiviral treatment are summarized in supplemental Table S2. Interestingly, the mRNA levels of SPHK1 in liver tissues were reduced after eradication of HCV in all 12 patients analyzed and HCV eradication significantly reduced the mRNA level of SPHK1 (Fig. 2b). This suggests that HCV infection itself could enhance the mRNA level of SPHK1; however, replication of HCV in Huh-7 cells did not alter the mRNA levels of SPHK1 and SPHK2 or the protein expression of SPHK1 (supplemental Fig. S2), indicating that liver inflammation or fibrosis caused by HCV infection could augment the mRNA level of SPHK1, unlike HCV proteins or viral RNA. Collectively, the analysis of human liver tissues suggests that SPHK1 plays an important role in the progression of liver fibrosis.

### Effects of peretinoin on mRNA levels of sphingolipid-related genes *in vitro*

**Figure 3:**
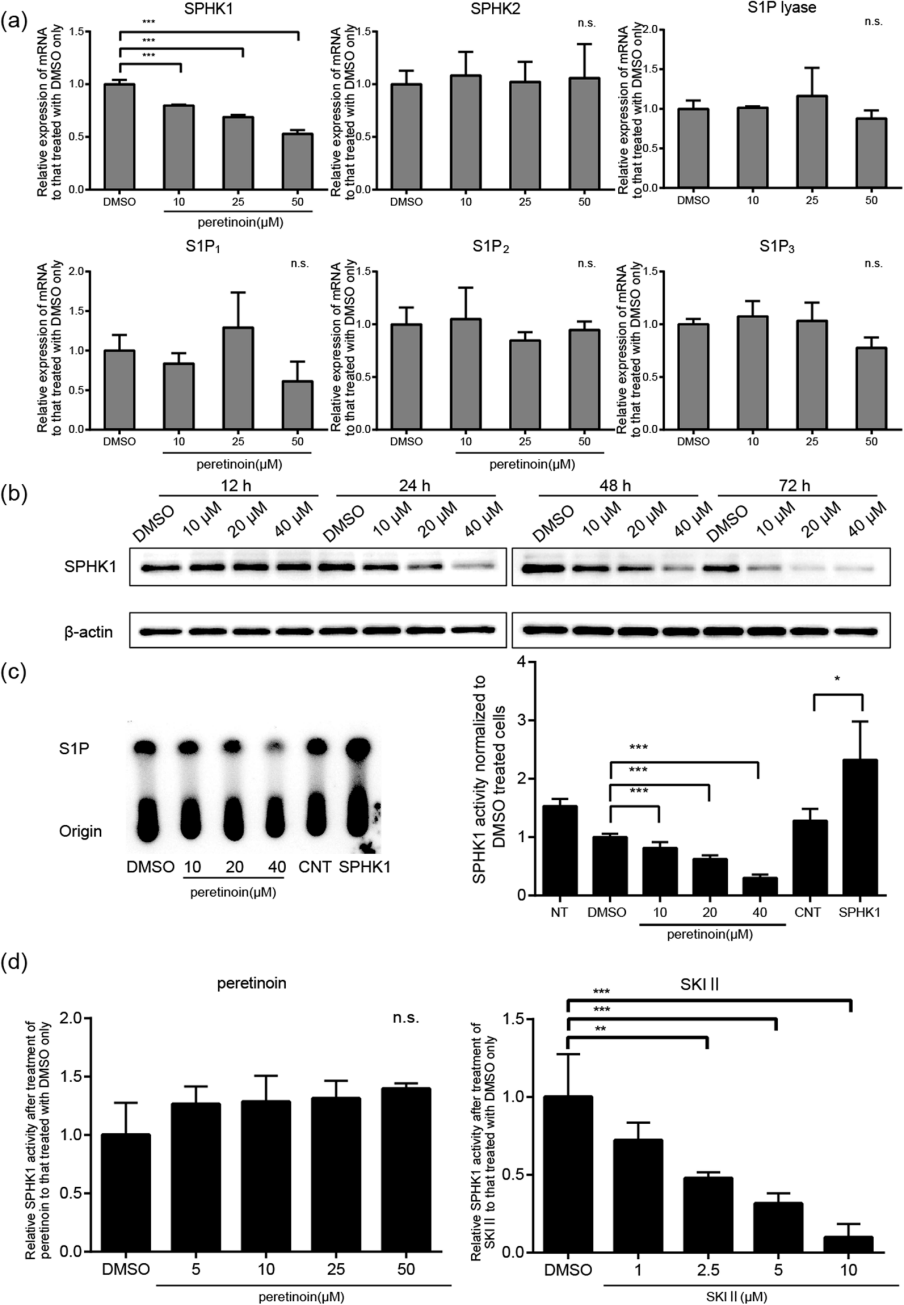
Effects of peretinoin on the mRNA level, protein expression, and enzymatic activity of SPHK1. (**a**) effects of peretinoin on the mRNA level of sphingolipid-related genes in Huh-7 cells. Huh-7 cells were treated with peretinoin at 10, 25, or 50 μM or with 0.5% DMSO for 72 h and total cellular RNA was extracted. The mRNA levels of SPHK1, SPHK2, S1P lyase, S1P receptor 1–3 (S1P_1_, S1P_2_, and S1P_3_), and β-actin were quantitated by qRT-PCR (SYBR green assay), and the mRNA levels of SPHK1, SPHK2, S1P lyase, S1P_1_, S1P_2_, and S1P_3_ were normalized to that of β-actin. The relative mRNA level of each gene under each condition was normalized to that of DMSO control. (**b**) Effects of peretinoin on the protein expression of SPHK1 in Huh-7 cells. Huh-7 cells were treated with 0.5% DMSO or peretinoin 10, 20, or 40 μM. Total cell lysates were collected 12, 24, 48, and 72 h later and then probed by western blotting with anti-SPHK1 and β-actin antibodies. Full-length gels and blots before cropping are shown in supplemental Fig. S8. (**c**) Effects of peretinoin on the enzymatic activity of SPHK1 in Huh-7 cells. Huh-7 cells were treated with 0.5% DMSO or peretinoin at 10, 20, and 40 μM and a plasmid encoding SPHK1 cDNA or an empty vector were transfected into Huh-7 cells. Total cell lysates were collected 72 h after initiation of peretinoin treatment and transfection and used in an *in vivo* SPHK activity assay. Synthesized S1P was visualized by using radioisotopes. The radioactive spots corresponding to S1P were scraped into vials and the radioactivity was measured in a scintillation counter and normalized to that of DMSO-treated cells or empty vector-transfected cells. Error bars indicate the standard deviation from three experiments and the statistical significance of the difference in the average was analyzed by one-way ANOVA. (**d**), effects of peretinoin on the enzymatic activity of SPHK1 *in vitro*. Human recombinant SPHK1 was incubated with DMSO or peretinoin at 5, 10, 25, or 50 μM or with SKI II at 5, 10, 25, or 50 μM as described in the Experimental procedures. Synthesized S1P was quantitated by a fluorescence-based method. The SPHK activity in each condition was normalized to that of DMSO control. *p < 0.05, **p < 0.01, ***p < 0.005.

We examined the effect of peretinoin on the mRNA levels of several crucial genes related to S1P metabolism and S1P receptors, such as SPHK1, SPHK2, S1P lyase, and S1P receptors 1–3 (S1P_1–3_) in Huh-7 cells. We treated Huh-7 cells with peretinoin at 10, 25, and 50 μM for 48 h, collected total RNA, and then determined the mRNA level by qRT-PCR. Although peretinoin did not show any effect on the mRNA level of SPHK2, S1P lyase, or S1P_1–3_, the mRNA level of SPHK1 was significantly reduced in a dose-dependent manner (Fig. 3a). Next, we treated Huh-7 cells with peretinoin at 10, 20, and 40 μM for 12, 24, 48, and 72 h and then measured SPHK1 expression by western blotting. We found that peretinoin exhibited suppressed SPHK1 expression after 24 h treatment, even at 10 μM. This suppression was more prominent after 72-h peretinoin treatment (Fig. 3b). A similar suppressive effect of peretinoin on the mRNA level of SPHK1 was also observed with another human hepatoma cell line, Hep3B (supplemental Fig. S3).

### Effects of peretinoin on the activity of SPHK1 *in vivo* and *in vitro*

To examine the effect of peretinoin on the enzymatic activity of SPHK1 *in vivo*, Huh-7 cells were treated with peretinoin at 10, 20, and 40 μM for 48 h. The cells were also transfected with a plasmid encoding human SPHK1 with N-terminal Flag tag and an empty vector, and we confirmed efficient expression of SPHK1 using western blotting (supplemental Fig. S4). We collected cell lysates from these cells and then determined *in vivo* SPHK1 activity by using ^32^P-labeled sphingosine as a substrate. While overexpression of SPHK1 boosted SPHK1 activity, peretinoin significantly inhibited SPHK1 activity in a dose-dependent manner (Fig. 3c). Because SPHK1 activity is enhanced in a post-transcriptional manner by various growth factors and cytokines^29^, we examined the effect of peretinoin on SPHK1 activity itself *in vitro* by using recombinant human SPHK1 protein. Although a well-known SPHK1 and 2 inhibitor, SKI II, inhibited SPHK1 activity in a dose-dependent manner, peretinoin did not show any inhibitory effect on SPHK1 activity up to 50 μM (Fig. 3d). Collectively, peretinoin is likely to possess the ability to reduce SPHK1 expression and activity, primarily by suppressing its transcription.

### Effects of peretinoin on the promoter activity of SPHK1

**Figure 4:**
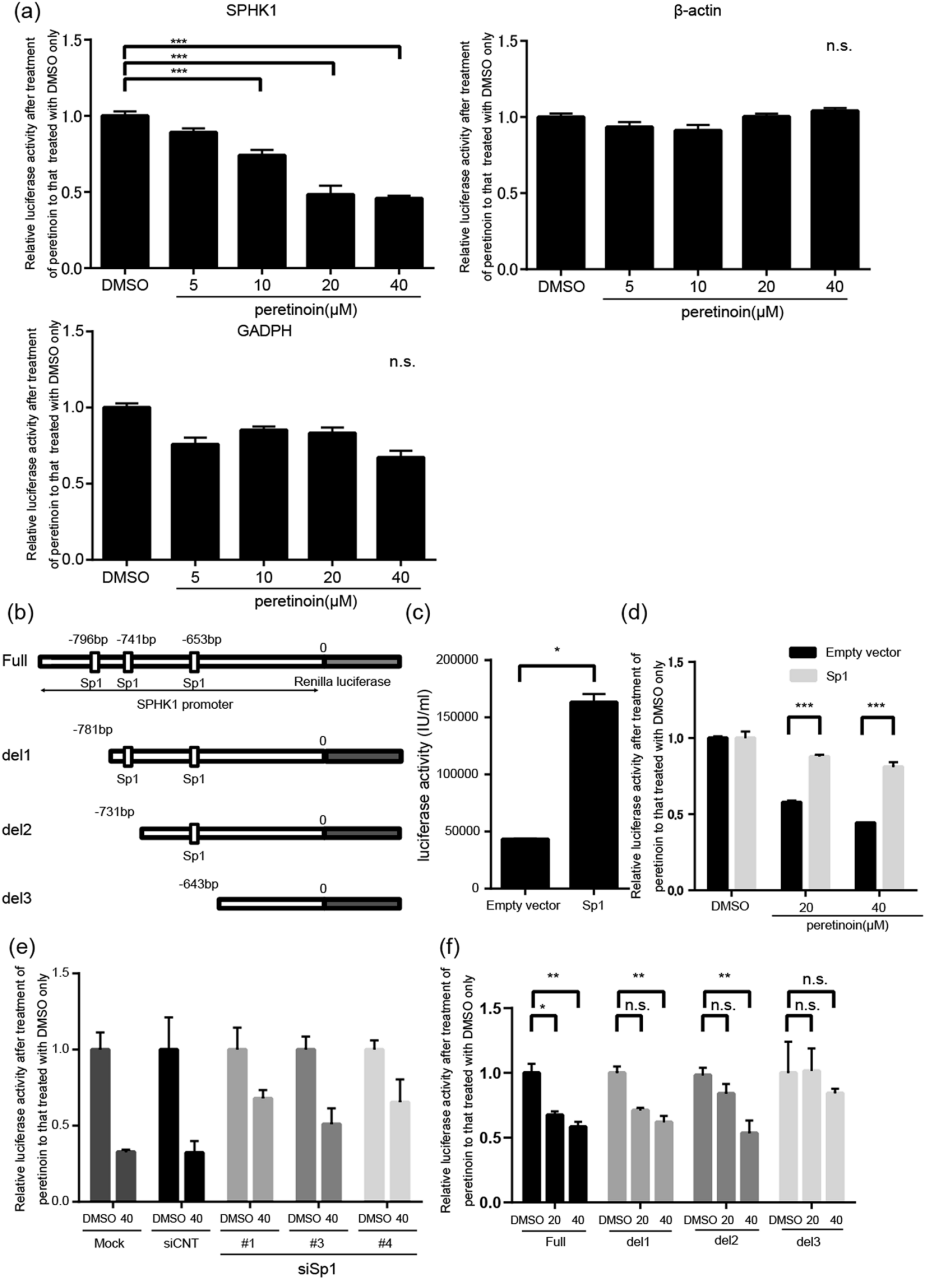
Effects of peretinoin on SPHK1 promoter activity. A effects of peretinoin on the promoter activity of SPHK1 and SPHK2. (**a**) Plasmids encoding luciferase under the control of the promoter region of SPHK1, SPHK2, β-actin, and GAPDH were transfected into Huh-7 cells and, 24 h later, DMSO or peretinoin at 1, 5, 10, 20, or 40 μM was added. After 48-h DMSO or peretinoin treatment, luciferase activity was determined. The luciferase activity in each condition was normalized to that of DMSO control. Error bars indicate the standard deviation from three experiments and the statistical significance of the difference in the average was analyzed by one-way ANOVA. (**b**) Possible binding sites of Sp1 in the SPHK1 promoter region and a representation of deletion mutants. This figure shows the possible binding sites of Sp1 in the promoter region of SPHK1 and the series of deletion mutants analyzed. (**c**) Enhancement of the promoter activity of SPHK1 by Sp1 overexpression. A plasmid encoding Sp1 or a control empty vector was co-transfected with a plasmid encoding luciferase under the control of the SPHK1 promoter region and, 48 h later, luciferase activity was determined. Error bars indicate the standard deviation from three experiments and the statistical significance of the difference in the average was analyzed by the Student’s t test. (**d**) Restoration of the suppressive effects of peretinoin on the promoter activity of SPHK1 by Sp1 overexpression. A plasmid encoding Sp1 or a control empty vector was transfected into Huh-7 cells. After 24 h, the cells were transfected with a plasmid encoding luciferase under the control of the promoter region of SPHK1 or a control vector without any promoter regions. After another 24 h, DMSO or peretinoin at 20 or 40 μM was added. After 48-h DMSO or peretinoin treatment, luciferase activity was determined. This figure shows the relative ratio of the luciferase activity from peretinoin-treated cells to that of DMSO-treated cells in cells transfected with the plasmid encoding Sp1 or the control empty vector. Error bars indicate the standard deviation from three experiments and the statistical significance of the difference in the average was analyzed by the Student’s t test. (**e**), attenuation of the suppressive effects of peretinoin on the promoter activity of SPHK1 by Sp1 knockdown. Three kinds of siRNA to Sp1, control siRNA (siCNT), or mock were transfected at 20 nM into Huh-7 cells and, 48 h later, the cells were transfected with the plasmid encoding luciferase under the control of the promoter region of SPHK1 or a control vector without any promoter regions. After another 24 h, DMSO or peretinoin at 40 μM was added. After 48-h DMSO or peretinoin treatment, luciferase activity was determined. This figure shows the relative ratio of the luciferase activity of cells treated with 40 μM peretinoin to that of DMSO-treated cells for each siRNA or mock transfection. (**f**), effects of deletion of the Sp1 binding site on the promoter activity of SPHK1. The plasmids encoding luciferase under the control of various lengths of promoter regions of SPHK1 were transfected into Huh-7 cells and, 24 h later, DMSO or peretinoin at 20 or 40 μM was added. After 48-h DMSO or peretinoin treatment, luciferase activity was determined. This figure shows the relative ratio of the luciferase activity of peretinoin-treated cells to that of DMSO-treated cells for each plasmid. Error bars indicate the standard deviation from three experiments and the statistical significance of the difference in the average was analyzed by two-way ANOVA. *p < 0.05, **p < 0.01, ***p < 0.005.

Next, to clarify how peretinoin modulates transcription of SPHK1, we investigated the effect of peretinoin on the transcriptional activity of SPHK1 by a conventional promoter assay. For this purpose, we used a plasmid encoding a promoter region of SPHK1. This plasmid contained 801 base pairs upstream of the start site for SPHK1 translation initiation and 211 base pairs downstream of the start site for SPHK1 translation initiation. We examined the effect of peretinoin on the promoter activity of SPHK1 as well as that of β-actin and GAPDH in Huh-7 cells (Fig. 4a). While peretinoin did not show any effects on the promoter activity of β-actin and GAPDH, it significantly suppressed the promoter activity of SPHK1 in a dose-dependent manner, suggesting that the suppressive effect of peretinoin on promoter activity would be specific for SPHK1.

### Transcriptional regulation of SPHK1 by peretinoin through Sp1

When we searched for possible transcription factor binding sites in the promoter region of SPHK1 by using a web-based method (TFBIND), we found three potential binding sites for Sp1, whose expression is suppressed by peretinoin^16^ (Fig. 4b). We confirmed the peretinoin suppression of Sp1 using western blotting (supplemental Fig. S5a). Therefore, we hypothesized that peretinoin could suppress SPHK1 transcription by inhibiting Sp1 expression. The transfection of a Sp1-encoding plasmid into Huh-7 cells efficiently increased Sp1 expression (supplemental Fig. S5b) and overexpression of Sp1 significantly boosted the activity of the SPHK1 promoter (Fig. 4c), suggesting crucial roles for Sp1 in SPHK1 promoter activity. We examined whether Sp1 overexpression could compensate for the peretinoin suppression of the promoter activity of SPHK1. When Sp1 was overexpressed in Huh-7 cells before treatment with 20 or 40 μM peretinoin, Sp1 overexpression canceled suppressive effect of peretinoin on the promoter activity to a significant extent (Fig. 4d).

We also investigated whether peretinoin could suppress the activity of SPHK1, even when the expression of Sp1 was already repressed. To suppress the expression of Sp1, we designed three different siRNA species targeting Sp1, and we confirmed their suppressive effects in Huh-7 cells by western blotting. Although it was difficult to efficiently knockdown the expression of Sp1 with one kind of siRNA, at least 50% reduction was achieved by each siRNA (supplemental Fig. S5c). Peretinoin treatment at 40 μM reduced the promoter activity of SPHK1 by about 80% compared with cells transfected with control siRNA and mock-transfected cells. On the other hand, the suppressive effect of peretinoin was significantly alleviated if the expression of Sp1 was knocked down by siRNA (Fig. 4e), indicating that suppression of SPHK1 promoter activity by peretinoin depends on Sp1 protein expression. Finally, to confirm the specific effect of Sp1 on the promoter activity of SPHK1, we made three deletion mutants of the SPHK1 promoter region that lacked the three possible Sp1 binding sites shown in Fig. 4B. While peretinoin still showed suppressive effects on the promoter activity of SPHK1 for deletion 1 and deletion 2, the suppressive effect of peretinoin was lost for deletion 3 (Fig. 4f), indicating that the Sp1 binding site closest to translation initiation, that is, the site of the deletion 3, would be the crucial one for the regulation of SPHK1 transcription by Sp1 while the other two potential binding sites would have little, if any, function.

### Effects of SPHK1 on hepatocarcinogenesis in DEN-induced mouse hepatoma models

**Figure 5:**
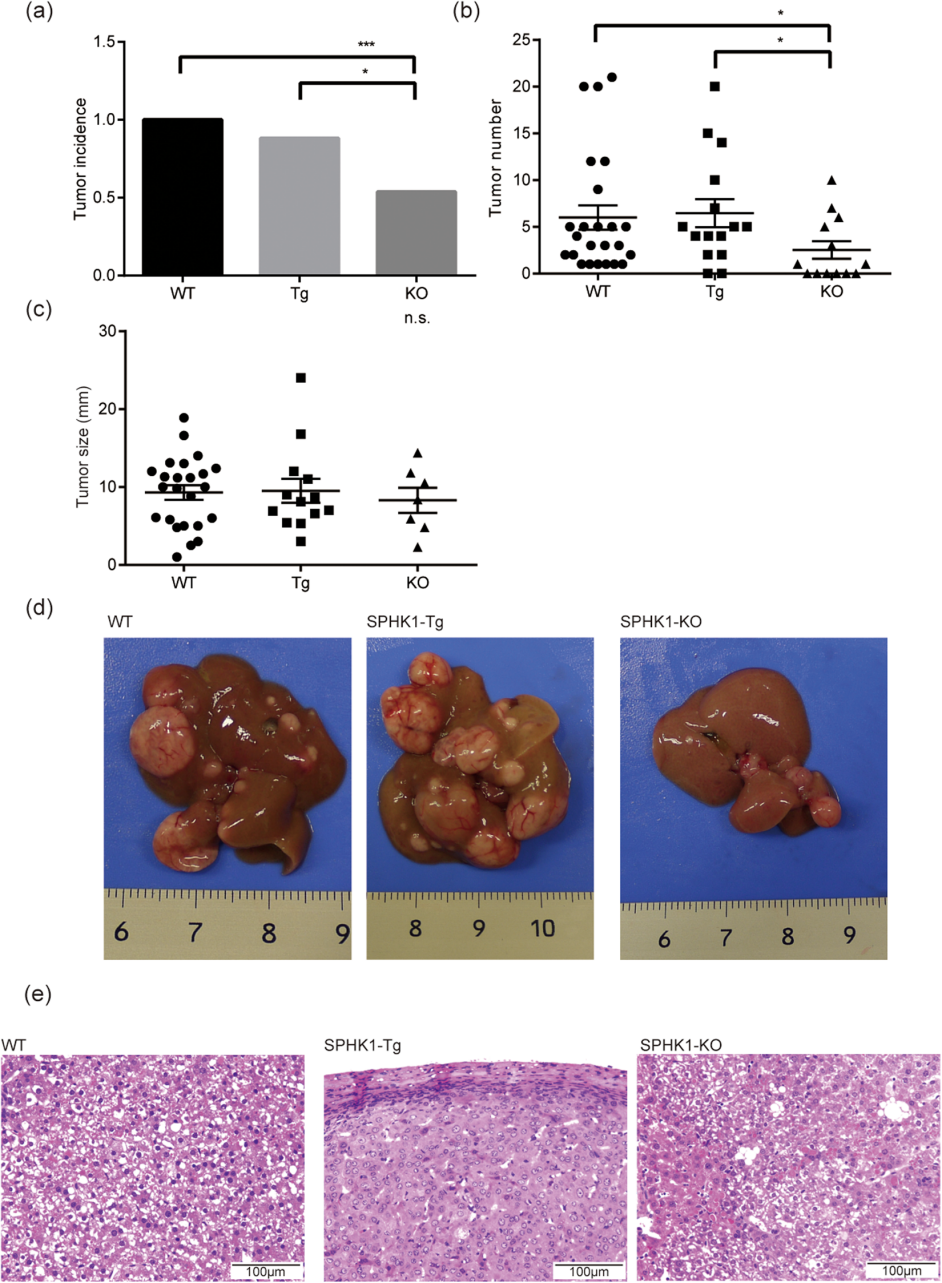
Effects of SPHK1 knockout on hepatocarcinogenesis in a DEN-induced hepatoma mouse model. DEN was injected into the peritoneal cavity of 2-week-old SPHK1 knockout, SPHK1 Tg, and wild-type mice at 25 mg/kg, and then those mice were sacrificed at 40 weeks old. (**a**) The numbers of mice that had a liver tumor at sacrifice. (**b**) The numbers of liver tumors in each mouse. (**c**) The maximum liver tumor size in individual mice that developed hepatoma. (**d**) Macrophotographs of representative mouse livers at sacrifice. (**e**) High power field microphotographs of representative mouse liver tumors stained with hematoxylin and eosin. Error bars shows the standard deviation from at least 20 mice, and the statistical significance of the difference in the average between these two groups was analyzed by the Student’s t test. *p < 0.05, ***p < 0.005

Our data suggest that peretinoin possesses a suppressive effect on SPHK1 transcription, which could decrease S1P production. Because the SPHK1–S1P axis promotes carcinogenesis in many cancers^19–22^, peretinoin could prevent hepatocarcinogenesis by suppressing SPHK1 transcription. However, because the exact roles of SPHK1 in hepatocarcinogenesis remain unclear, we analyzed SPHK1 transgenic and knockout mice under DEN-induced hepatocarcinogenesis conditions. Although all of the wild-type mice and 86% of the SPHK1 transgenic mice developed a liver tumor, only 53% of the SPHK1 knockout mice developed liver tumors (Fig. 5a). The frequency and numbers of liver tumors per knockout mouse were significantly lower than those of the wild-type and transgenic mice (Fig. 5b,d)). Comparison of the maximum liver tumor size among the wild-type, SPHK1 transgenic, and knockout mice suggested that liver tumors in SPHK1 knockout mice were generally smaller than those in the SPHK transgenic mouse, although this difference was not statistically significant (Fig. 5c). We also compared pathological features of liver tumors from wild-type, SPHK1 transgenic, and SPHK1 knockout mice. The liver tumor cells from wild-type and SPHK1 knockout mice had almost similar size of nucleus to that of non-tumorous liver cells frequently accompanied with Mallory body and moderate fatty change, and these liver tumors were diagnosed as well-differentiated HCC. On the other hand, the liver tumor cells from SPHK1 Tg mice showed nuclear atypia with higher tumor cell density and milder fatty change than wild-type and SPHK1 knockout mice, and these liver tumors were diagnosed as moderately differentiated HCC (Fig. 5e and supplemental Fig. S7a). The non-tumorous liver tissues from these mice showed similar pathological features, such as fatty change, Mallory body, and no evident fibrosis; however, infiltration of inflammatory cells in portal area from SPHK1 Tg mice was stronger than that from wild-type and SPHK1 knockout mice (and supplemental Fig. S7b). These results strongly indicate that SPHK1 plays an important role in hepatocarcinogenesis induced by DEN, as well as tumor progression.

## Discussion

Peretinoin (generic name code: NIK-333) is an orally available acyclic retinoid. Administration of peretinoin significantly reduces the incidence of post-therapeutic HCC recurrence and improves the survival rate of patients in clinical trials^13–15^. Larger-scale clinical studies are ongoing in various countries to validate its clinical efficiency in HCC prevention after curative treatment. Retinoids generally exert their biological functions by binding to one of two distinct nuclear receptors, RAR and RXR, both of which have three subtypes (α, β, and γ)^30,31^. Both RAR and RXR, as transcription factors, also modulate the expression of many retinoid-related genes.

How peretinoin prevents hepatocarcinogenesis has been extensively studied by using mouse models and hepatoma cell lines. For example, by using a PDGF-C transgenic mouse, our group showed that peretinoin repressed the development of hepatic fibrosis and tumors and that this effect seemed to be due to inhibition of the PDGF signaling pathway underlying fibrogenesis, angiogenesis, and Wnt/β-catenin^16^. Kagawa *et al*.^32^ reported that peretinoin inhibited hepatocarcinogenesis in the rat induced by DEN through suppression of transforming growth factor-α (TGF-α) expression and cell proliferation. Other studies showed that peretinoin suppressed cell growth and induced apoptosis in hepatoma cells and a DEN-induced mouse hepatoma model through inactivation of the Ras-ERK signaling system and subsequent RXRα dephosphorylation^18,33–35^. In addition, peretinoin can modulate many proteins and pathways important for cell proliferation and carcinogenesis, such as cyclin D1^36^, fibroblast growth factor receptor 3^37^, the cell cycle inhibitor protein p21^38^, Krüppel-like factor 5 (KLF5)^39^, telomerase^40^, the MAP kinase pathway^41^, and the autophagy pathway^17^.

In addition to these reported mechanisms, our present results suggest that peretinoin could prevent hepatocarcinogenesis through transcriptional suppression of SPHK1. Although the SPHK–S1P axis is involved in many aspects of cancer progressions, the specific areas modulated by SPHK1 should be clarified in future studies. Analysis of the SPHK1 promoter region in this study suggested that SPHK1 transcription would be regulated by the Sp1 transcription factor and that peretinoin would suppress the transcription of SPHK1 through downregulation of Sp1 (Fig. 4). How peretinoin suppresses Sp1 expression should be addressed in future studies. We also examined the effects of other retinoids, such as all-trans retinoic acid, 9-cis retinoic acid, and 13-cis retinoic acid, on mRNA levels of SPHK1, finding that these retinoids also possessed the ability to suppress SPHK1 transcription even at lower concentrations where peretinoin did not show suppressive effects on SPHK1 expression (Supplemental Fig. S6). These data suggest that this suppressive effect is universally shared among retinoids and that the suppressive effect of peretinoin seems weaker than the other retinoids tested. These results indicate that the preventive effect of peretinoin on hepatocarcinogenesis may not completely depend on suppressive effect on SPHK1, because peretinoin was reported to show a stronger anti-proliferative effect in Huh-7 cells than others retinoids such as all-trans retinoic acid, 13-cis retinoic acid, and 9-cis retinoic acid^42^.

Many lines of evidence show that S1P promotes cancer by modulating certain factors. Therefore, SPHK1 and SPHK2, two enzyme essential for S1P generation, as well as S1P itself and S1P receptors, have been recognized as promising targets for cancer treatment. Many SPHK inhibitors, either selective or non-selective for SPHK1 and SPHK2, have been developed, and some of them have entered clinical trials. For example, safingol, an inhibitor of both SPHK1 and SPHK2, was tested in phase I clinical trials for advanced solid cancers and showed anti-cancer effects without any serious adverse effects when used with cisplatin^23^. Recently, the efficacy and safety of a specific SPHK2 inhibitor, ABC294640, was also shown in phase I clinical trials for patients with advanced solid cancers^24^. A monoclonal antibody to S1P, sphingomab, was also developed, and it reduced tumor growth and showed anti-angiogenic effects in animal models^25^. In addition to SPHK inhibitors and S1P antibodies, S1P receptor antagonists could also suppress S1P function. In liver diseases, FTY720, which is a potent antagonist of S1P receptor and approved for use in patients with multiple sclerosis, was reported to suppress the proliferation of liver-derived cancer cells^43,44^.

However, the importance of the SPHK–S1P axis in the development of liver fibrosis and hepatoma is less well understood than for other cancers. Nonetheless, crucial roles of the SPHK–S1P axis in liver fibrosis and hepatoma have recently been reported via analysis of human samples. Sato *et al*.^28^ showed that the mRNA levels of SPHK1, but not those of SPHK2, were increased in livers with severe fibrosis compared with those with mild fibrosis; however, S1P was not increased in severe fibrotic liver tissue. Uranbileg *et al*.^45^ reported that the mRNA levels of SPHK1, SPHK2, and S1P lyase were increased in HCC tissues compared with adjacent non-HCC tissues; however, S1P levels were unexpectedly lower in HCC tissues than in non-HCC tissues. Li *et al*.^46^ also demonstrated that SPHK1 mRNA, but not SPHK2 mRNA, and S1P were increased in severe fibrotic liver, irrespective of the cause of the liver fibrosis. The same group also showed that S1P induced the expression of angiopoietin 1 mRNA through S1P receptors 1 and 3 in hepatic stellate cells in liver fibrosis mouse models^47^. These studies indicate that SPHK1 in liver tissues with fibrosis is generally upregulated, whereas the changes in SPHK2 and S1P differ from study to study. These data suggest that the SPHK–S1P axis plays certain important roles in the progression of liver fibrosis, hepatocarcinogenesis, and hepatoma.

In the present study, we compared the mRNA levels of SPHK1 in HCV-infected human liver between two groups, those with severe liver fibrosis and those with mild liver fibrosis, finding that the SPHK1 mRNA level was significantly higher in severe fibrosis than in mild fibrosis (Fig. 2). The mRNA level of SPHK1 was also increased in the liver in two different liver fibrosis-hepatoma mouse models (Fig. 1). These results suggest that SPHK1 plays a crucial role in liver fibrosis progression, which is consistent with previous studies on fibrotic liver. Interestingly, elimination of HCV from the infected liver by antiviral treatment resulted in a significant reduction in SPHK1 mRNA within the liver. We also showed that HCV replication did not increase the expression of SPHK1 protein, suggesting that liver inflammation due to HCV, rather than HCV viral proteins or RNA, could augment the level of SPHK1 mRNA. Recently developed HCV treatments involving direct-acting antivirals can eliminate HCV in more than 95% of patients, and amelioration of liver fibrosis would be expected after HCV elimination due to the subsequent reduction in SPHK1.

In the present study, SPHK1 knockout mice developed liver tumors in response to DEN treatment significantly less frequently than wild-type mice, strongly suggesting crucial roles of SPHK1 in hepatocarcinogenesis. HCC generally arises from chronic liver diseases, inflammation, or liver fibrosis caused by factors such as viral hepatitis, alcohol, and fat. Although we showed crucial roles for SPHK1 in a DEN-induced hepatocarcinogenesis model in this study, further validation is needed in different mouse models of liver fibrosis and hepatoma.

The present study could not identify the main target of peretinoin, liver fibrosis or hepatocarcinogenesis. The roles of SPHK–S1P have been more intensively studied in liver fibrosis than in hepatocarcinogenesis. S1P was first shown to stimulate rat hepatic stellate cell proliferation, suggesting that S1P could be a pro-fibrotic factor in the liver^48^; several studies clearly showed that this pro-fibrotic effect is regulated thorough the S1P receptors S1P_1_, S1P_2_, and S1P_3_ on hepatic stellate cells^46,49–52^, suggesting that depletion of S1P by SPHK inhibitors could be effective for liver fibrosis. SPHK in tumor cells may not be a promising treatment target for cancer, because novel, highly potent, and selective SPHK inhibitors that reduced S1P to undetectable levels in tumor cells did not affect their growth *in vivo* or *in vitro* ^53–55^. Because hepatoma generally develops from liver fibrosis, SPHK inhibitors are highly expected to inhibit hepatocarcinogenesis through suppression of liver fibrosis. While these two steps are constitutive, and difficult to separate, the effects of peretinoin on liver fibrosis should be tested separately from those on hepatocarcinogenesis.

In the present study, we showed that peretinoin can suppress SPHK1 transcription, reducing SPHK1 protein levels and SPHK1 activity. Furthermore, by using SPHK1 knockout mice, we demonstrated the crucial roles of SPHK1 in hepatocarcinogenesis. Collectively, our results indicate that the SPHK–S1P axis is a promising target in the treatment of liver fibrosis and hepatocarcinogenesis. We therefore plan to perform intensive studies of the roles of the SPHK–S1P axis in liver diseases in order to develop effective treatments targeting the SPHK–S1P axis.

## Methods

### Reagents and materials

A mammalian expression plasmid of N-terminal myc-FLAG-tagged human SPHK1 (GenBank data base accession number NM_021972) and one encoding Sp1 under the control of cytomegalovirus promoter were purchased from OriGene (Rockville, MD). Three kinds of siRNAs targeting Sp1 (FlexiTube GeneSolution GS6667 SI#00150983, 02648065, and 02648072) were purchased from Qiagen (Hilden, Germany), as well as non-targeting siRNA (siGENOME Non-Targeting siRNA Pool #2, #D-001206-14-50) from Thermo Fisher Scientific (Waltham, MA). Peretinoin was kindly provided by Kowa Company (Aichi, Japan). Sphingosine kinase inhibitor 2 (SKI II, formally 4-[[4-(4-chlorophenyl)-2-thiazolyl]amino]-phenol) was purchased from Cayman Chemical Company (CAS No 312636-16-1; Ann Arbor, MI). These compounds were dissolved in DMSO. All final dilutions contained 0.5% DMSO. DEN was purchased from Sigma-Aldrich (St. Louis, MO).

### Cells

Huh-7 cells, a human hepatoma cell line, were maintained in DMEM (Thermo Fisher Scientific) supplemented with 10% fetal bovine serum (Thermo Fisher Scientific), 1% L-glutamine (Thermo Fisher Scientific), and 1% penicillin/streptomycin (Thermo Fisher Scientific) in a humidified atmosphere of 5% CO_2_ at 37 °C.

### Transfection

Plasmid and siRNA transfection was performed according to the manufacturers’ instructions by using X-tremeGENE HP DNA Transfection Reagent (Roche, Basel, Switzerland) and Lipofectamine RNAiMAX (Thermo Fisher Scientific), respectively.

### *In vivo* SPHK activity assay

Sphingosine kinase activity was measured as described previously^56^. Briefly, the labeled S1P was separated by TLC on Silica Gel G-60 (GE Healthcare, Little Chalfont, UK) with 1-butanol/ethanol/acetic acid/water (80:20:10:20, v/v) and visualized by autoradiography. The radioactive spots corresponding to S1P were scraped into vials and the radioactivity was measured in a scintillation counter.

### *In vitro* SPHK activity assay

A sphingosine kinase 1 inhibitor screening assay kit was purchased from Cayman Chemical Company. Briefly, a fluorescence-based method was adopted to screen the ability of peretinoin to act as an SHPK1 inhibitor. Human recombinant SPHK1, along with ATP, phosphorylates D-erythro-sphingosine C-18, and then S1P and ADP are produced. Next, ADP helps to synthesize hydrogen peroxide (H_2_O_2_) in a series of enzymatic reactions performed according to the manufacturer’s protocol. H_2_O_2_ in the presence of 10-Acetyl-3, 7-dihydroxyphenoxazine yields their fluorescent product, resorufin. Resorufin was analyzed with an excitation wavelength between 530 and 540 nm and an emission wavelength between 580 and 590 nm.

### Western blotting

Western blotting and immunostaining were performed as described previously^57,58^. Briefly, cells were washed in PBS and lysed in a radioimmunoprecipitation assay buffer containing complete Protease Inhibitor Cocktail and PhosSTOP (Roche). The membranes were blocked in Blocking One or Blocking One-P solution (Nacalai Tesque, Kyoto, Japan), and the expression of SPHK1 was evaluated with rabbit anti-SPHK1 (#A302-177A; Bethyl Laboratories, Montgomery, TX), rabbit anti-Sp1 (#5931; Cell Signaling Technology, Danvers, MA), and rabbit β-actin antibody (#4967; Cell Signaling Technology).

### Quantitative real-time PCR

Total RNA was isolated from cell samples using a GenElute Mammalian Total RNA Miniprep Kit (Sigma-Aldrich). cDNA was synthesized by using a High-Capacity cDNA Reverse Transcription Kit (Thermo Fisher Scientific), and the abundance of target RNAs in cDNA was quantitated by TaqMan assay or SYBR green assay. For the TaqMan assay, primer pairs and probes for human SPHK1 and β-actin were obtained from the TaqMan assay reagent library of Thermo Fisher Scientific, and the abundance of each RNA was determined as described previously^59^. For the SYBR green assay, qRT-PCR was performed on a CFX384 machine (Bio-Rad, Hercules, CA) using SYBR Green Master Mix (Thermo Fisher Scientific). The sequences of primers used for real-time PCR are listed in supplemental Table S1 for human SPHK1, SPHK2, S1P lyase, S1P_1_, S1P_2_, and S1P_3_, and β-actin. The abundance of each RNA was determined by using the comparative threshold cycle method^60^.

### Reporter assay

Reporter plasmids containing the Renilla luminescent reporter gene and the human promoter regions of SPHK1 and β-actin were purchased from Active Motif (Carlsbad, CA). Huh-7 cells were transfected with 500 ng of each reporter construct by using X-tremeGENE HP DNA Transfection Reagent (Thermo Fisher Scientific) according to the manufacturer’s protocol. After 24-h incubation, the cells were treated with peretinoin or transfected with an Sp1-coding plasmid or siRNA for Sp1. Subsequently, the cells were cultured for 48 h, and cell lysates were used to measure luciferase reporter gene expression using a GloMax-Multi + Detection System (Promega, Madison, WI).

### Prediction of Sp1-binding sites within the SPHK1 promoter region

The human genomic DNA sequence was obtained from the National Center for Biotechnology Information Entrez Gene (http://www.ncbi.nlm.nih.gov/entrez/query.fcgi?db=gene). Potential Sp1-binding sites were sought using TFBIND with a threshold of 0.9 (http://tfbind.hgc.jp/). The threshold represents the minimum probability of predicted transcription factors.

### Mouse experiments

SPHK1 knockout mice^61^ and SPHK1 transgenic mice^62^ were kindly provided by Dr. Pu Xia (University of Sydney) and Dr. Mark A. Febbraio (Baker IDI Heart and Diabetes Institute), respectively. To induce liver tumors, 25 mg/kg DEN was injected into the peritoneal cavity of these mice and wild-type mice at 2 weeks of age^63^; the mice were then sacrificed at 40 weeks of age. The protocol of mouse experiments was approved by Kanazawa University (approval #AP-163781) and mouse experiments were carried out in accordance with the Guidelines for the Care and Use of Laboratory Animals of the Takaramachi Campus of Kanazawa University, Japan.

### Histopathology

Mouse liver tissues were fixed in 10% formalin and stained with hematoxylin and eosin. A certified pathologist histopathologically analyzed the images based on WHO Classification of Tumours of the Digestive System (2010, 4^th^ edition^64^).

### Human samples

The present study evaluated 178 patients with chronic liver diseases due to HCV at the Graduate School of Medicine at Kanazawa University Hospital (Kanazawa, Japan) and its related hospitals in Japan. The clinical characteristics of 168 of the patients have been described previously^65^ and we included 10 new patients. All patients were treated with pegylated interferon α-2b and ribavirin combination treatment for 48 weeks and had undergone liver biopsy before the combination treatment. We also included another 12 patients with chronic liver diseases due to HCV from the Graduate School of Medicine at Kanazawa University Hospital; they had undergone liver biopsy prior to antiviral treatment, as well as after successful viral elimination. Total liver RNA was extracted as described previously^65^ and used for further analyses. Informed consent was obtained from all patients, and ethics approval for the study was obtained from the Ethics Committee for Human Genome/Gene Analysis Research at Kanazawa University Graduate School of Medical Science. In addition, all experiments were performed in accordance with relevant guidelines and regulations.

### Statistics

The results are expressed as the mean ± standard deviation. Significance was defined as p < 0.05 and was tested by the Student’s t test or paired t test. Statistical analyses were performed using GraphPad Prism 6.05 (La Jolla, CA, USA).

## Electronic supplementary material

Supplemental Information
