# Supplementary material for: Peretinoin, an acyclic retinoid, inhibits hepatocarcinogenesis by suppressing sphingosine kinase 1 expression in vitro and in vivo
Source: Sci Rep. 2017 Dec 5;7:16978. doi: 10.1038/s41598-017-17285-2 (PMC5717167; doi:10.1038/s41598-017-17285-2)
Supplement: Supplementary file 1 — Supplemental Information [file 41598_2017_17285_MOESM1_ESM.doc]

**Supplemental Information**

**Peretinoin, an acyclic retinoid, inhibits hepatocarcinogenesis by suppressing sphingosine kinase 1 expression *in vitro* and *in vivo***

Masaya Funaki^1^, Juria Kitabayashi^1^, Tetsuro Shimakami^1^, Naoto Nagata^2^, Yuriko Sakai^2^, Kai Takegoshi^1^, Hikari Okada^1^, Kazuhisa Murai^1^, Takayoshi Shirasaki^1^, Takeru Oyama^3^, Taro Yamashita^1^, Tsuguhito Ota^2^, Yoh Takuwa^4^, Masao Honda^1^, and Shuichi Kaneko^1^

^1^Department of Gastroenterology, Kanazawa University, Kanazawa, Ishikawa, Japan

^2^Advanced Preventive Medical Sciences Research Center, Kanazawa University, Kanazawa, Ishikawa, Japan

^3^Department of Molecular and Cellular Pathology, Ishikawa, Japan

^4^ Department of Physiology, Kanazawa University School of Medicine, Kanazawa, Ishikawa, Japan

**A list of the materials included**

Supplemental Table S1

Supplemental Table S2

Supplemental Figure S1

Supplemental Figure S2

Supplemental Figure S3

Supplemental Figure S4

Supplemental Figure S5

Supplemental Figure S6

Supplemental Figure S7

Supplemental Figure S8

Table S1. Sequences of the primers used for the CYBR green assay

| Primer name | Sequence (5'-3') |
| --- | --- |
| hSK1_F | GGCGTCATGCATCTGTTCTA |
| hSK1_R | ACACACCTTTCCCATCCTTG |
| hSK2_F | TTTGCCCTCACCCTTACATC |
| hSK2_R | CAGAAGTAGGCCGCTGAGTC |
| hS1Plyase_F | CTTGATGCACTTCGGTGAGA |
| hS1Plyase_R | TCCACCCCTTAGCAGTCATC |
| hS1PR1_F | AACTGACCTCGGTGGTGTTC |
| hS1PR1_R | GCTACTCCTGCCAACAGGTC |
| hS1PR2_F | TCATCGTCATCCTCTGTTGC |
| hS1PR2_R | GCCAGAGAGCAAGGTATTGG |
| hS1PR3_F | ACCATCGTGATCCTCTACGC |
| hS1PR3_R | AGGCCACATCAATGAGGAAG |
| hβActin_F | TTCTACAATGAGCTGCGTGTG |
| hβActin_R | GGGGTGTTGAAGGTCTCAAA |

**Table S2. Characteristics of the 12 patients whose liver SPHK1 mRNA levels were measured before antiviral treatment and after SVR**

| **Patient number** | **Age at start of antiviral treatment (years)** | **Sex** | **Antiviral treatment** | **Elapsed time between liver biopsies prior to antiviral treatment and after SVR (days)** |
| --- | --- | --- | --- | --- |
| **1** | 76 | M | PEG-IFN+RBV | 658 |
| **2** | 59 | F | PEG-IFN+RBV | 751 |
| **3** | 67 | M | PEG-IFN+RBV | 412 |
| **4** | 38 | M | PEG-IFN+RBV | 1716 |
| **5** | 54 | M | PEG-IFN+RBV | 662 |
| **6** | 50 | F | PEG-IFN+RBV | 790 |
| **7** | 58 | F | PEG-IFN+RBV | 615 |
| **8** | 68 | M | PEG-IFN+RBV | 393 |
| **9** | 71 | F | PEG-IFN+RBV | 2719 |
| **10** | 68 | M | PEG-IFN+RBV | 597 |
| **11** | 63 | M | PEG-IFN+RBV | 525 |
| **12** | 46 | F | PEG-IFN+RBV | 812 |

| **Patient number** | **Relative expression of SPHK1 mRNA (prior to antiviral treatment)** | **Relative expression of SPHK1 mRNA (post-SVR)** | **METAVIR score (prior to antiviral treatment)** | **METAVIR score (post-SVR)** | **ALT level (prior to antiviral treatment)** | **ALT level (post-**  **SVR)** |
| --- | --- | --- | --- | --- | --- | --- |
| **1** | 4.24 | 3.34 | F4A1 | F4A1 | 25 | 12 |
| **2** | 3.78 | 2.39 | F2A2 | F2A1 | 47 | 11 |
| **3** | 2.17 | 1.17 | F2A1 | F3A1 | 88 | 19 |
| **4** | 3.11 | 2.89 | F1A1 | F1A2 | 120 | 42 |
| **5** | 1.94 | 1.48 | F1A2 | F1A1 | 56 | 20 |
| **6** | 1.01 | 0.72 | F1A1 | F1A0 | 45 | 13 |
| **7** | 2.34 | 1.25 | F3A2 | F3A1 | 362 | 23 |
| **8** | 3.37 | 1.68 | F3A2 | F3A1 | 93 | 26 |
| **9** | 4.45 | 2.37 | F2A2 | F4A1 | 159 | 93 |
| **10** | 1.84 | 1.54 | F1A1 | F1A1 | 171 | 12 |
| **11** | 1.99 | 0.82 | F1A1 | F1A1 | 46 | 15 |
| **12** | 9.58 | 2.09 | F4A2 | F4A1 | 57 | 20 |

ALT, alanine aminotransferase.

**Supplemental Figures**

**Figure S1**


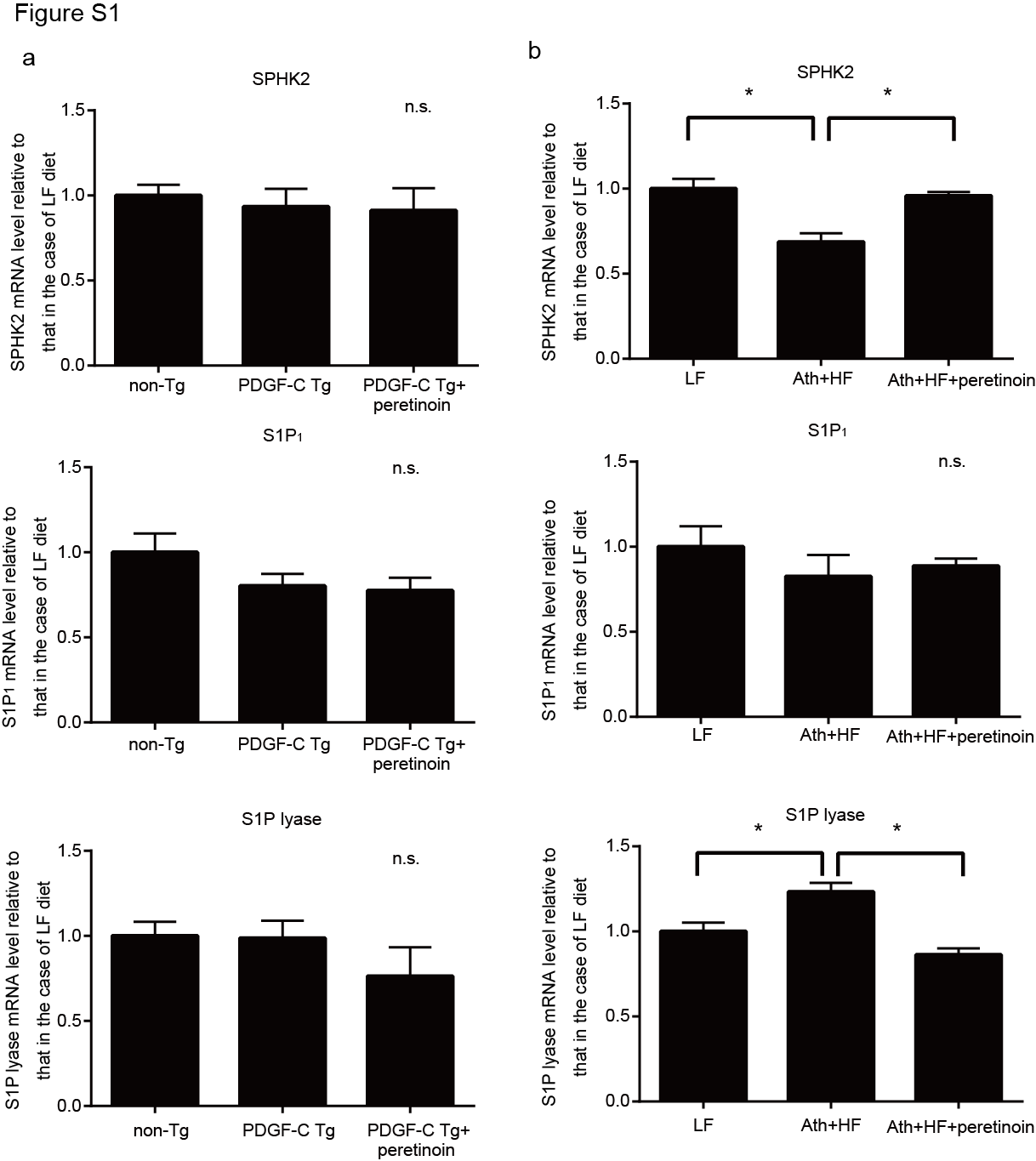


**Figure S1. Liver mRNA levels of SPHK2, S1P lyase, and S1P_1_ in the PDGFC-Tg and Ath-HF diet mouse models.** a, the mRNA level of SPHK1 in the liver of PDGF-C Tg mice treated with or without 0.06% peretinoin was normalized to that of non-Tg mice. The mRNA levels were calculated from our previously published microarray data^1^. This figure shows the relative mRNA levels of SPHK2, S1P lyase, and S1P_1_ under each condition to that of non-Tg mice. Error bars indicate the standard deviation from three mice. The statistical significance of the difference in the average between the two groups was analyzed by the Student’s t test. b, the mRNA level of SPHK1 in the liver of Ath-HF diet mice treated with or without 0.03% peretinoin was normalized to that of LF mice. The mRNA levels were calculated from our previously published microarray data^2^. This figure shows the relative mRNA levels of SPHK2, S1P lyase, and S1P_1_ under each condition to that of LF mice. Error bars indicate the standard deviation from three mice. Statistical significance was analyzed as above. *p < 0.05.

**Figure S2**


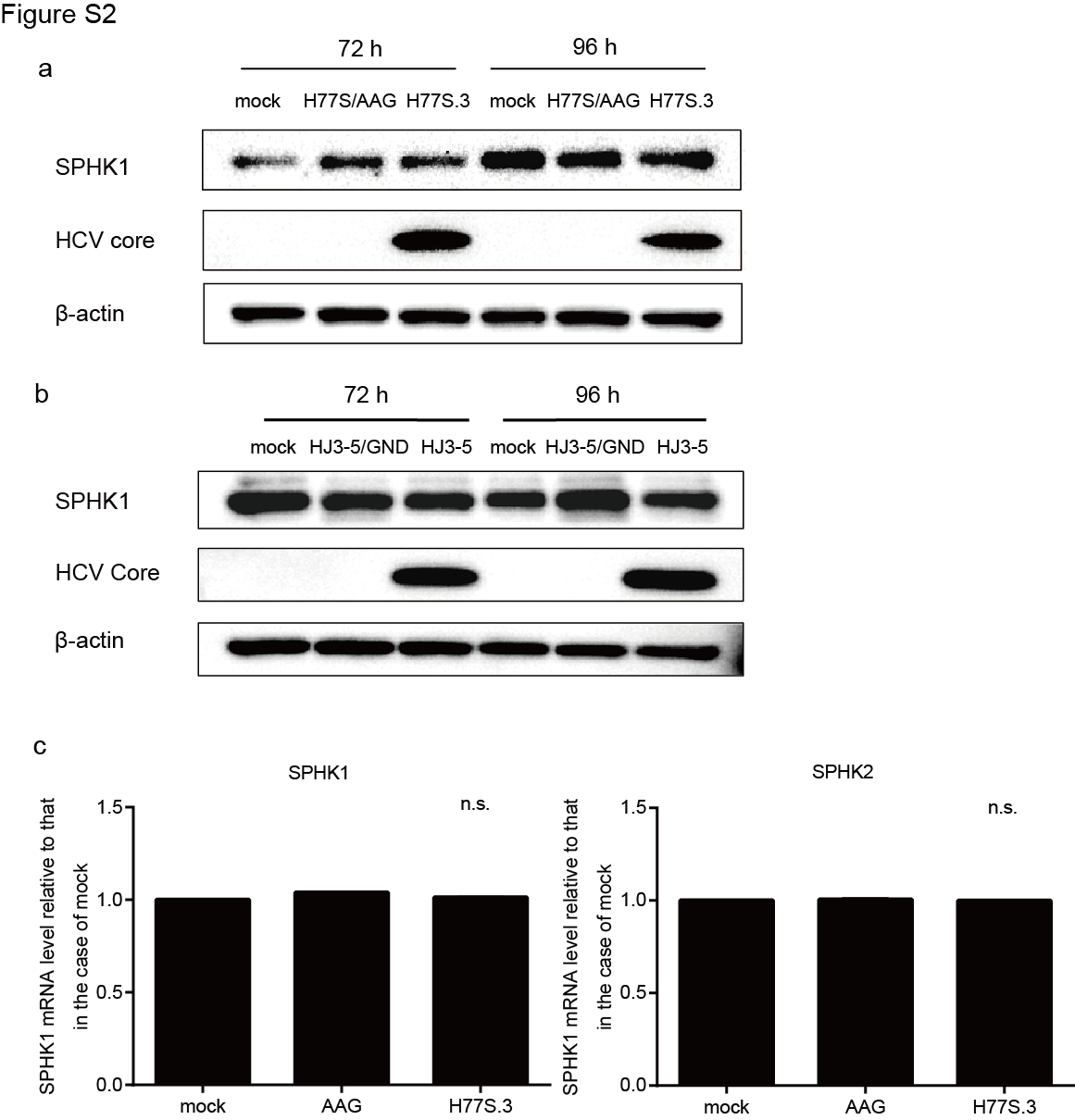


**Figure S2. HCV replication does not influence SPHK1 protein expression.** H77S.3 is an infectious clone for cell culture of genotype 1a, and H77S/AAG is a replication-incompetent mutant whose catalytic center of nonstructural protein 5B (NS5B) was mutated from GDD to AAG^3,4^. HJ3-5 is a chimeric clone of H77S and genotype 2a JFH-1, in which the sequence encoding core-NS2 of the genotype 1a H77c virus was placed within the background of the genotype 2a JFH-1 virus. HJ3-5/GND is a replication-incompetent mutant whose catalytic center of NS5B was mutated from GDD to GND^5,6^. HCV RNA was synthesized from the plasmid encoding these HCVs by in vitro transcription, purified, and transfected into cells by Trans-IT mRNA Transfection Reagent (Mirus Bio, Madison, WI) as described in our previous work^4^. a, HCV RNAs (H77S.3 and replication-defective H77S/AAG) were transfected into Huh-7 cells and, 72 and 96 h later, total cell lysates and total cellular RNA were collected. The expression of HCV core protein, SPHK1, and β-actin was probed by western blotting with the appropriate antibodies. b, HCV RNAs (HJ3-5 RNA and replication-defective HJ3-5/GND) were transfected into Huh-7 cells and, 72 and 96 h later, total cell lysates and total cellular RNA were collected. The expression of HCV core protein, SPHK1, and β-actin was probed by western blotting with the appropriate antibodies. c, Total cellular RNA was extracted from H77S.3, H77S/AAG, and mock-transfected cells 96 h after RNA transfection. The RNA levels of SPHK1, SPHK2, and β-actin were measured by qRT-PCR (TaqMan assay), with the mRNA levels of SPHK1 and SPHK2 normalized to those of β-actin. The relative mRNA levels were normalized to those from mock-transfected cells. Error bars show the standard deviation from three experiments, although they are almost invisible due to the small standard deviation.

**Figure S3**


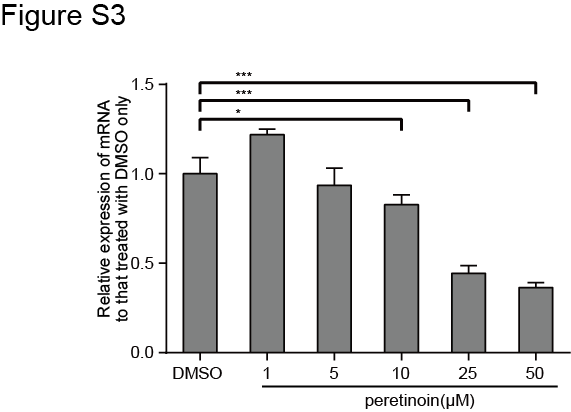


**Figure S3. Effects of peretinoin on the mRNA level of SPHK1 in Hep3B cells.** Hep3B cells were treated with peretinoin at 1, 5, 10, 25 or 50 μM or with 0.5% DMSO for 72 h and total cellular RNA was extracted. The mRNA level of SPHK1 was determined by qRT-PCR (TaqMan assay). The relative mRNA level under each concentration was normalized to that of DMSO control. Error bars indicate the standard deviation of three experiments and the statistical significance of the difference in the average was analyzed by one-way ANOVA. *p < 0.05, ***p < 0.005.

**Figure S4**


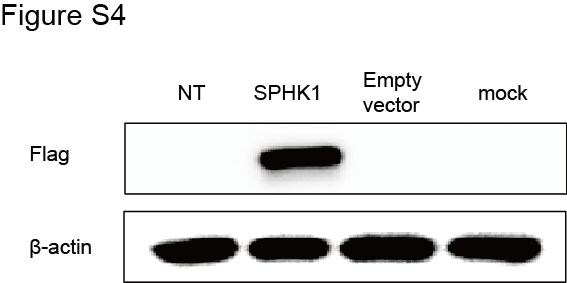


**Figure S4. Efficient expression of SPHK1 from a transected plasmid.** The plasmids encoding human SPHK1 with N-terminal Flag tag, an empty vector, and mock were transfected into Huh-7 cells. Efficient expression of SPHK1 was confirmed using western blotting with anti-Flag M2 antibody (catalog #1804; Sigma-Aldrich). NT, non-treatment.

**Figure S5**


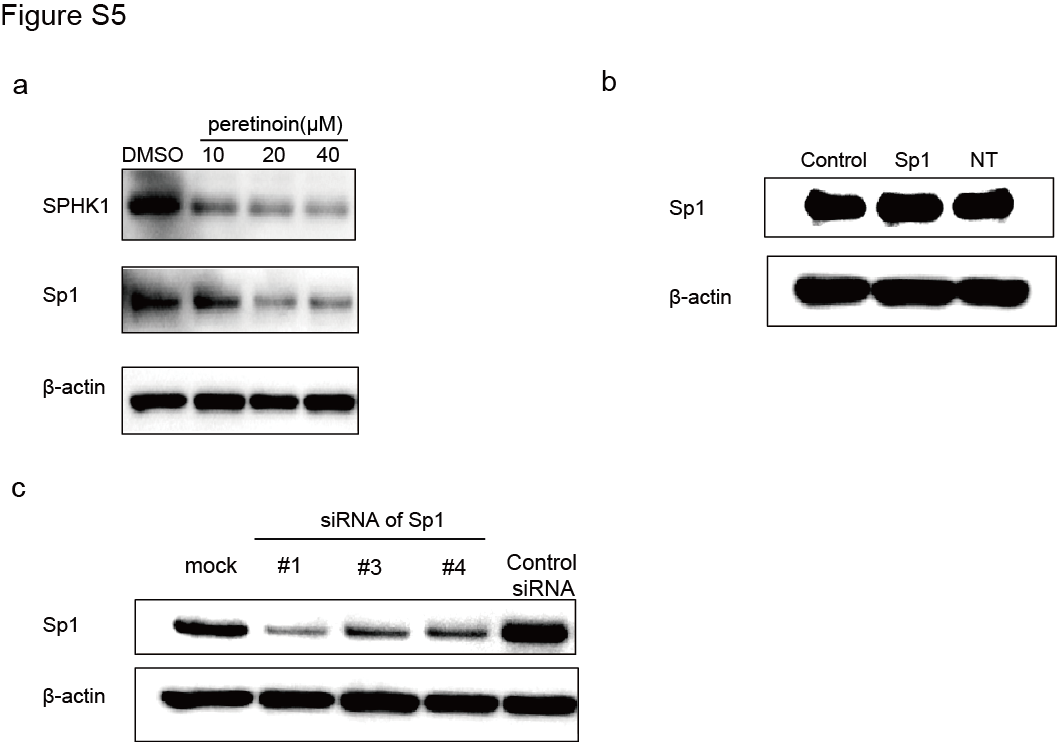


**Figure S5. Transcriptional regulation of SPHK1 by peretinoin through Sp1.** *a*, suppression of Sp1 by peretinoin. Huh-7 cells were treated with DMSO and peretinoin at 10, 20, or 40 μM for 48 h and cell lysates were collected. The expression of Sp1, SPHK1, and β-actin was probed by western blotting with the appropriate antibodies. *b*, Sp1 overexpression. A plasmid encoding cDNA of Sp1 and empty vector were transfected into Huh-7 cells and, 72 h later, cell lysates were collected. The expression of Sp1 and β-actin was probed by western blotting with the appropriate antibodies. Control, total cell lysates from the empty vector plasmid-transfected cells; NT, total cell lysates from untreated cells. *c*, Sp1 knockdown. Three siRNAs (#1, #2, and #3) targeting Sp1 and non-targeting siRNA (control siRNA) were separately transfected into Huh-7 cells at 20 nM using Lipofectamine RNAiMAX (Thermo Fisher Scientific) and, 72 h later, cell lysates were collected. The expression of Sp1 and β-actin was probed by western blotting with the appropriate antibodies. NT, non-treatment.

**Figure S6**


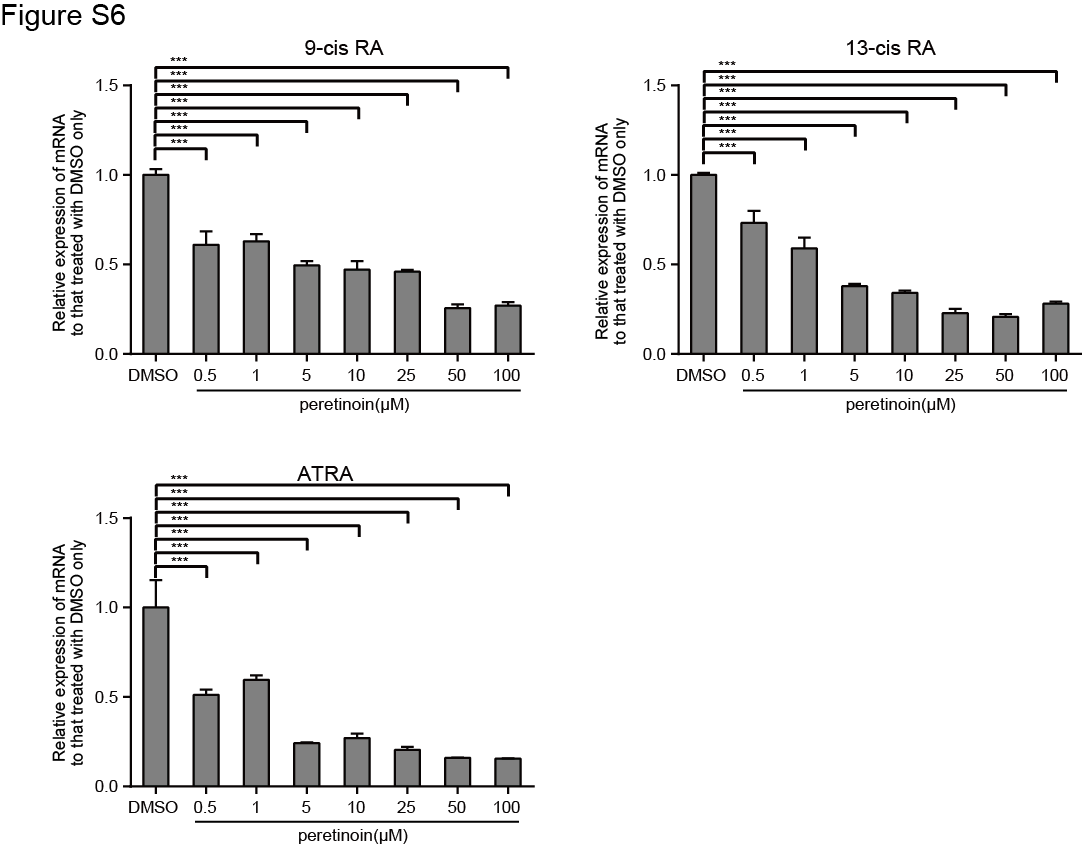


**Figure S6. Effects of other retinoids on the mRNA level of SPHK1.** Huh-7 cells were treated with 9-cis retinoic acid (9-cis RA), 13-cis retinoic acid (13-cis RA) or all-trans retinoic acid (ATRA) at 0.5,1, 5, 10, 25, 50 or 100 μM or with 0.5% DMSO for 72 h and total cellular RNA was extracted. The mRNA level of SPHK1 was determined by qRT-PCR (TaqMan assay). The relative mRNA level at each concentration was normalized to that of DMSO control. Error bars indicate the standard deviation of three experiments and the statistical significance of the difference in the average was analyzed by one-way ANOVA. ***p < 0.005.

**Figure S7**

**
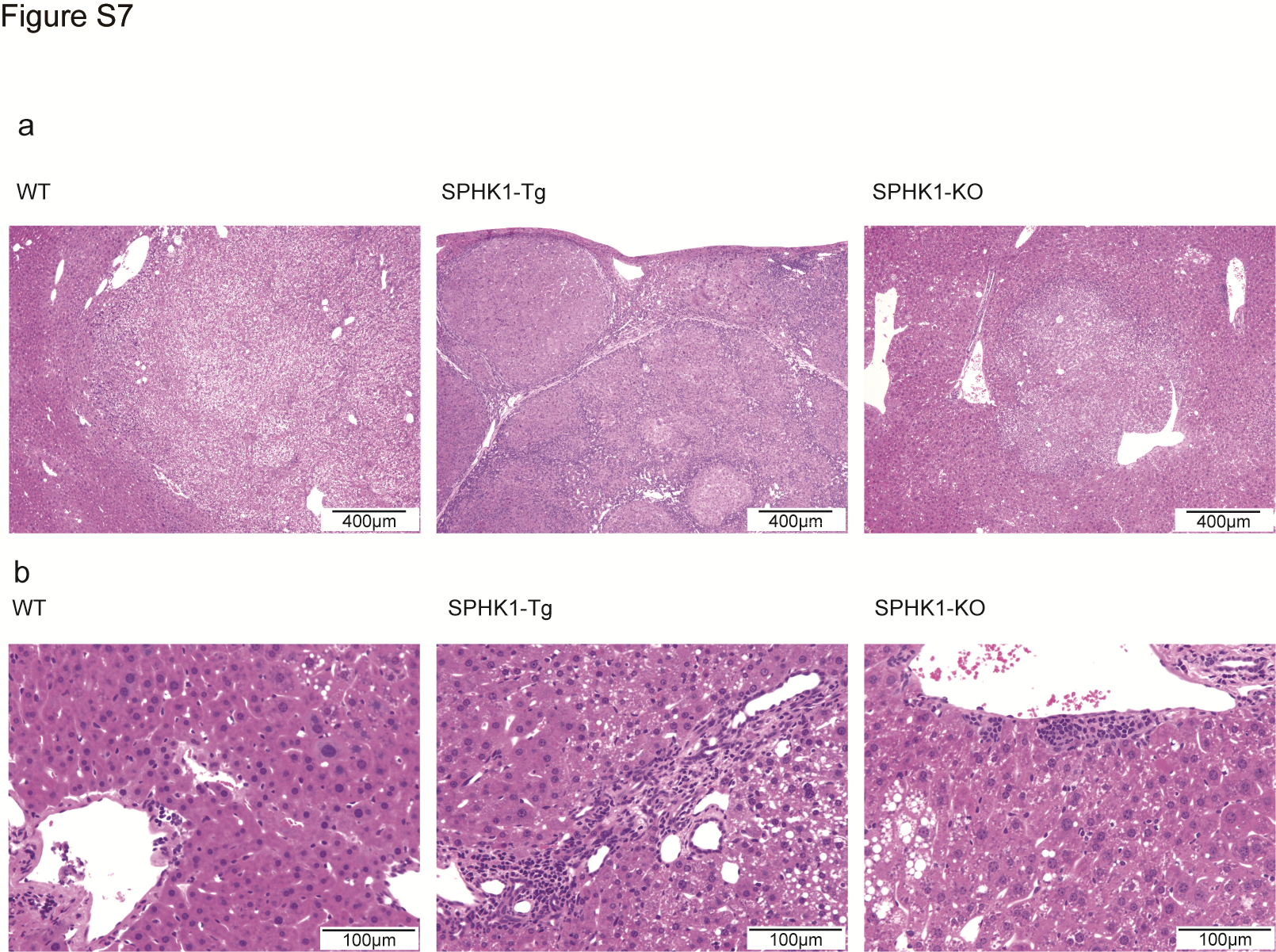
**

**Figure S7. Microphotograph of representative mouse liver tumors and non-tumorous liver.** DEN was injected into the peritoneal cavity of 2-week-old SPHK1 knockout, SPHK1 Tg, and wild-type mice at 25 mg/kg, and then those mice were sacrificed at 40 weeks old. a, low power field microphotographs of mouse liver tumors and non-tumorous liver b, high power field microphotographs of non-tumorous liver.

**Figure S8**


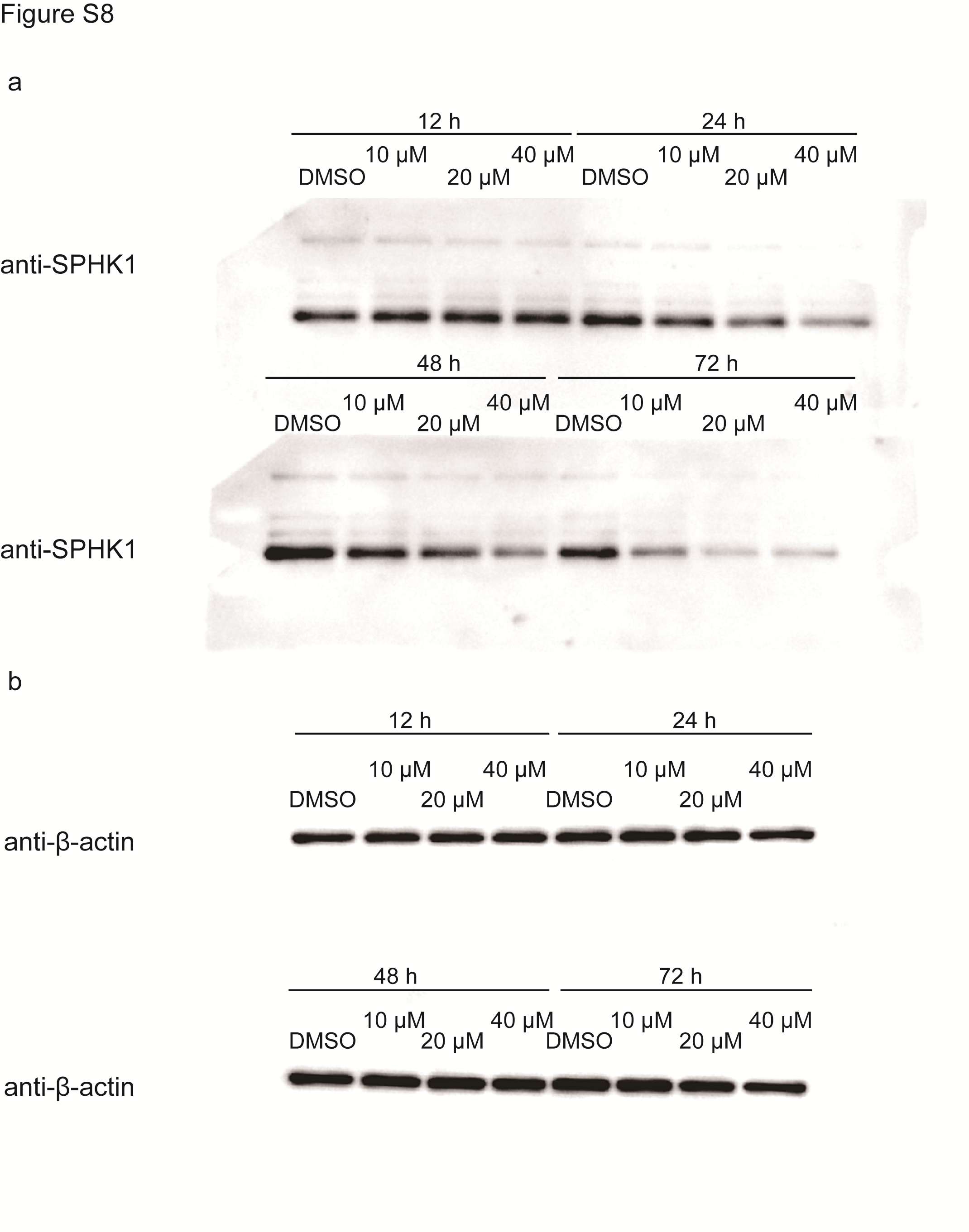


**Figure S8.** **Full-length gels and blots before cropping for Figure 3b.** Effects of peretinoin on the protein expression of SPHK1 in Huh-7 cells. Huh-7 cells were treated with 0.5% DMSO or peretinoin 10, 20, or 40 μM. Total cell lysates were collected 12, 24, 48, and 72 h later and then probed by western blotting with anti-SPHK1 and β-actin antibodies. Full-length gels and blots before cropping for Figure 3b were shown here. a, anti-SPHK1, b, anti-β-actin.

**References in Supplemental Information**

1 Okada, H. *et al.* Acyclic retinoid targets platelet-derived growth factor signaling in the prevention of hepatic fibrosis and hepatocellular carcinoma development. *Cancer Res* **72**, 4459-4471 (2012).

2 Okada, H. *et al.* Peretinoin, an acyclic retinoid, suppresses steatohepatitis and tumorigenesis by activating autophagy in mice fed an atherogenic high-fat diet. *Oncotarget*, **8**, 39978-39993 (2017).

3 Yi, M., Villanueva, R. A., Thomas, D. L., Wakita, T. & Lemon, S. M. Production of infectious genotype 1a hepatitis C virus (Hutchinson strain) in cultured human hepatoma cells. *Proceedings of the National Academy of Sciences of the United States of America* **103**, 2310-2315 (2006).

4 Shimakami, T. *et al.* Protease inhibitor-resistant hepatitis C virus mutants with reduced fitness from impaired production of infectious virus. *Gastroenterology* **140**, 667-675 (2011).

5 Ma, Y., Yates, J., Liang, Y., Lemon, S. M. & Yi, M. NS3 helicase domains involved in infectious intracellular hepatitis C virus particle assembly. *Journal of virology* **82**, 7624-7639 (2008).

6 Yi, M., Ma, Y., Yates, J. & Lemon, S. M. Compensatory mutations in E1, p7, NS2, and NS3 enhance yields of cell culture-infectious intergenotypic chimeric hepatitis C virus. *Journal of virology* **81**, 629-638 (2007).
